# Brief report: attitudes and barriers toward genetic testing in pleural mesothelioma: a nationwide Italian survey

**DOI:** 10.1007/s10689-026-00596-7

**Published:** 2026-07-24

**Authors:** Luigi Cerbone, Matteo Perrino, Federica Grosso, Monica Ganzinelli, Giuseppe Lo Russo, Giulia Pasello, Paolo Andrea Zucali, Giovanni Luca Ceresoli, Mario Occhipinti, Marika Sculco, on the behalf of MET-I (Mesothelioma team Italy)

**Affiliations:** 1 SSD Mesotelioma, Melanoma E Tumori Rari, AOU SS Antonio E Biagio E Cesare Arrigo Alessandria, Alessandria, Italy; 2 Department for Sustainable Development and Ecological Transition, Università del Piemonte Orientale, Vercelli, Italy; 3 Medical Oncology Department, IRCCS Humanitas Research Hospital, Milan, Italy; 4 Medical Oncology 1, Fondazione IRCCS Istituto Nazionale Dei Tumori, Milan, Italy; 5 Medical Oncology 2, Istituto Oncologico Veneto, Padua, Italy; 6 Department of Surgery, Oncology and Gastroenterology, University of Padova, Padua, Italy; 7 Department of Biomedical Sciences, Humanitas University, Pieve Emanuele, Milan, Italy; 8 Humanitas Gavazzeni, Bergamo, Italy; 9 Department of Health Sciences, Università del Piemonte Orientale, Novara, Italy

**Keywords:** Mesothelioma, germline testing, somatic DNA testing, BAP1 tumor predisposition syndrome

## Abstract

Pleural mesothelioma (PM) is a malignancy with a relevant genetic component, with germline mutations identified in up to 12% of cases. International guidelines recommend universal germline testing; however, its implementation in routine clinical practice remains inconsistent. This nationwide survey aimed to assess current clinical practice, attitudes, and barriers related to genetic testing among Italian specialists within the Mesothelioma Team Italy (MET-I) network. A 24-item questionnaire was distributed to MET-I members in 2025, exploring access to testing, clinical use, and perceived obstacles to both germline and somatic DNA sequencing. Forty-five physicians responded (53.6%). Although access to germline testing was relatively high (77.8%), only 6.7% of respondents routinely recommended it. Conversely, somatic NGS was widely available (95.6%) and primarily used for clinical trial screening (66.7%). The main barriers to germline testing included costs (62.2%), limited access to genetic counseling (51.1%), and insufficient physician awareness (48.8%). Despite the availability of genetic testing technologies, germline genetic testing in PM is underutilized in Italy.

## Introduction

Pleural mesothelioma (PM) is a rare, asbestos-related malignancy originating from the pleura [1]. It is characterized by large chromosomal rearrangements followed by the accumulation of point mutations, leading to the inactivation of tumor suppressor genes such as BAP1, NF2, and CDKN2A/B [2]. Germline pathogenic mutations have been associated with PM development in up to 12% of cases [3]. While the most widely studied germline pathogenic mutations involve BAP [1, 4] generally in the context of the BAP1 tumor predisposition syndrome, germline alterations in homologous recombination repair (HRR) genes—particularly ATM, BRCA1, and BRCA2—have also been implicated in PM development [5–7]

The identification of germline mutations in mesothelioma patients has important clinical implications, including recognition of hereditary cancer predisposition syndromes, genetic counseling for family members, and development of tailored surveillance strategies. The 2025 American Society of Clinical Oncology (ASCO) guidelines for PM recommend germline DNA testing for all patients. In addition, ASCO guidelines state that insufficient evidence is available to routinely recommend somatic DNA testing in all pleural mesothelioma patients despite acknowledging the potential prognostic implications and predictive relevance in the context of targeted agents clinical trials [8]. Nevertheless, clinical practice in countries outside the United States may vary substantially due to differences in healthcare organization, access to genetic counseling, and sequencing technologies. Indeed, Italian guidelines for the diagnosis and treatment of pleural mesothelioma, although acknowledging that this disease has a strong genetic component, do not formally advise to perform germline testing in these patients [9]. The aim of this study was to evaluate the attitudes, clinical practices, and perceived barriers related to germline and somatic genetic testing among Italian physicians within the Mesothelioma Team Italy (MET-I) network, a multidisciplinary national network dedicated to the study and management of mesothelioma.

## Methods

A 24-item multiple-choice questionnaire was developed by the Research Working Group of MET-I, pilot-tested by four participants, and distributed via email on March 4, 2025 to all MET-I members, with a completion deadline of July 31, 2025. The survey was administered using Google Forms; participation was voluntary and responses were anonymized. No patient-related data were collected; accordingly, formal ethical approval was not required per institutional policies.

The questionnaire comprised four sections: demographic and clinical practice data (7 items); germline testing attitudes (6 items); somatic testing attitudes (6 items); and perceived barriers and potential improvements (5 items). Data were collected in Microsoft Excel and analyzed descriptively.

**Fig. 1:**
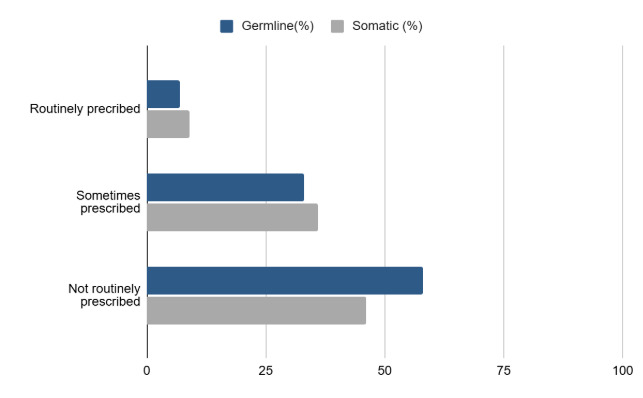
Prescribing habits for genetic testing in mesothelioma, number of assessed participants = 45

**Fig. 2:**
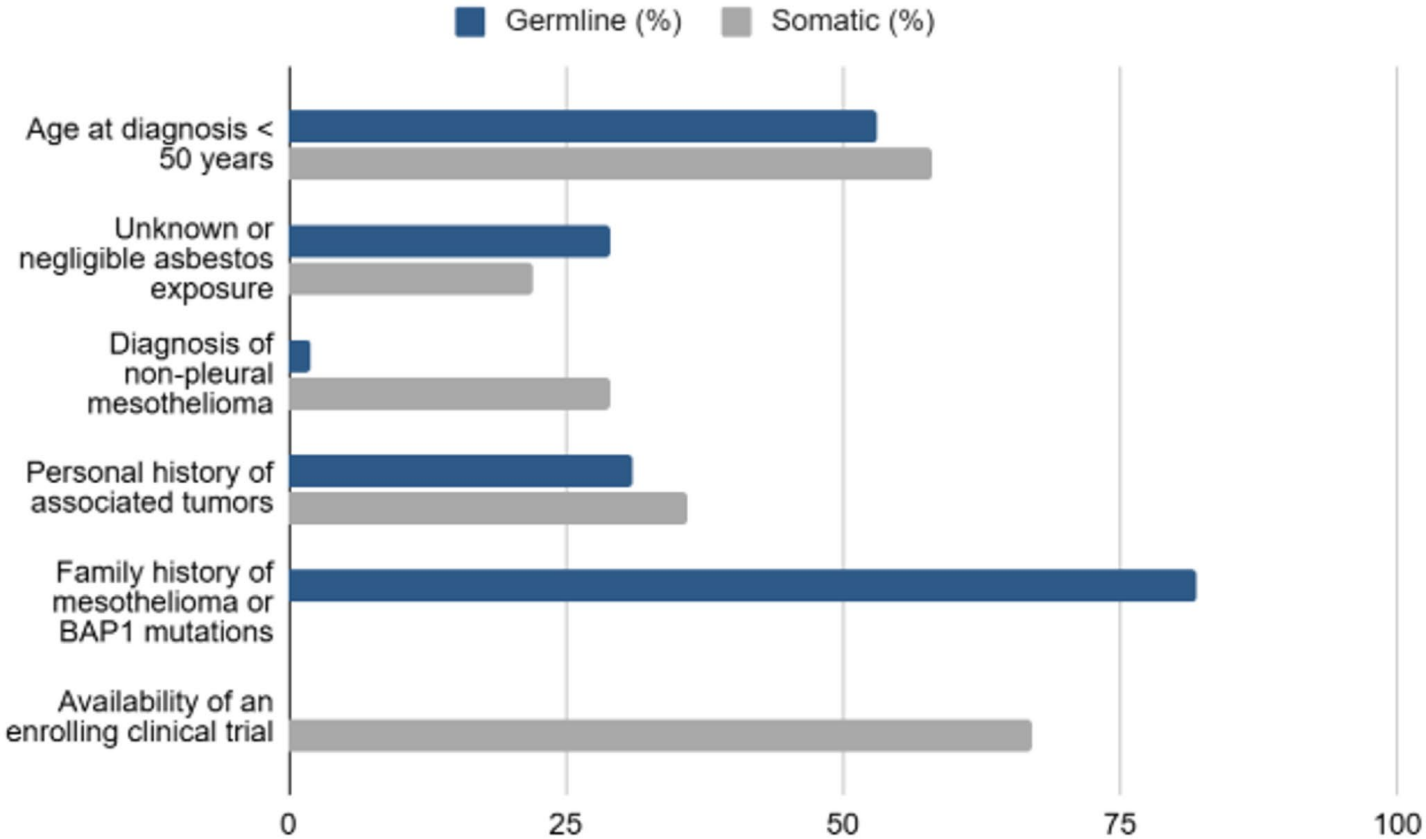
Reason for prescription of germline vs somatic DNA testing in mesothelioma

## Results

Of 84 MET-I members surveyed, 45 (53.6%) responded. Respondents were predominantly female (60%), medical oncologists (66.7%), aged 30–45 years (66.7%) and working in Northern Italy (88.9%) at academic centers (84.4%) (Table 1).

**Table 1:** Characteristics of Survey Participants

Characteristic	Number [total = 45] (%)
*Gender*	
Female	27(60%)
Male	18(40%)
*Medical specialty*	
Medical oncology	30(66.7%)
Pathology	5(11.1%)
Radiation oncology	3(6.7%)
Thoracic surgery	2(4.4%)
Pulmonology	2(4.4%)
Other	3(6.7%)
*Years of practice*	
0–5 years	10(22.2%)
6–10 years	10(22.2%)
11–20 years	18(40%)
> 21 years	7(15.6%)
*Geographic distribution*	
North	40(88.9%)
Central Italy	2(4.4%)
Southern Italy & Islands	3(6.7%)
*Type of institution*	
Academic hospital	38(84.4%)
General hospital/other	7(15.6%)

### Germline DNA testing

Access to germline testing was available at 77.8% of institutions, with BAP1 multigene panels being the most common test (40%), followed by monogenic testing (24.4%). Only 22.2% of respondents had formal training in genetic counseling. Despite this availability, most participants (57.8%) never routinely prescribed germline testing, 35.6% did so occasionally, and only 3 (6.7%) prescribed it routinely. The main indications cited were family history of mesothelioma and known familial pathogenic variants (both 82.2%). Other triggers included family history of mesothelioma-associated tumors (68.9%), age < 50 years at diagnosis (53.3%), and family history of unrelated tumors or personal history of mesothelioma-associated tumors (31.1%)(Figs. 1, 2). 

### Somatic DNA testing

Somatic NGS was widely available, with only 4.4% of respondents lacking access. The most common platform was a panel with > 50 genes (66.7%). Most participants did not routinely recommend somatic testing (55.6%), while 35.5% did so occasionally and 8.8% routinely. The most common reason for prescribing somatic testing was the availability of a clinical trial with molecular screening criteria (66.7%), followed by age < 50 years (57.8%) and good performance status after standard treatment failure (35.5%). Somatic NGS results influenced clinical practice occasionally 37.8%, sometimes 28.9%, often 15.6% and never 17.8% of respondents (Figs. 1, 2).

### Barriers and proposed solutions

The main barriers to genetic testing implementation were cost (62.2%), limited access to genetic counseling (51.1%), lack of physician awareness (48.8%), and lack of guidelines (31.1%). Proposed improvements included better education (60%), improved access to testing (55.5%), enhancement of institutional policies (53%), and insurance coverage (17.8%). The majority of respondents (86.7%) expressed interest in further education, preferring webinars (60%), written guidelines (53.3%), online courses (44.4%), and in-person workshops (42.2%).

## Discussion

This survey, the first to assess genetic testing practices in PM within a national Italian expert network, reveals a significant gap between the availability of germline testing and its actual clinical implementation. Despite 77.8% of respondents having access to germline testing, only 6.7% prescribed it routinely. Several factors likely contribute to this disconnect. First, Italy currently lacks formal national indications for germline testing in mesothelioma, leaving prescription decisions to individual physician judgment. Second, while germline pathogenic mutations might predispose to mesothelioma development, asbestos exposure is still needed to develop the disease, even if a lower extent. This might mislead physicians in considering mesothelioma an exposure-only related disease, resulting in a missed prescription of a germline test. Third, limited access to dedicated genetic counseling services and low physician awareness of testing criteria further hinder implementation. Fourth, a reported factor leading to a reduced prescription of germline testing in mesothelioma is patient refusal due to concerns regarding discrimination related to employment, insurance, and asbestos-related litigation [10]. This is relevant in the United States, where asbestos is still employed and asbestos related litigations are on an individual basis. Nevertheless, in Italy, asbestos is definitively banned since 1992 and compensation for asbestos exposure is provided in every mesothelioma patient with a recognized asbestos exposure by INAIL (Istituto Nazionale Assistenza Infortuni sul Lavoro). Finally, regional variability in reimbursement policies within the Italian healthcare system likely contributes to heterogeneous practices.

Somatic NGS testing, by contrast, appears more integrated into routine practice, primarily driven by clinical trial eligibility rather than standard decision-making. This reflects a real-world mismatch with current ASCO guidelines not routinely recommending genetic testing despite its potential prognostic and predictive implications. Notably, classically actionable genomic alterations are uncommon in PM [11], and recent trials such as the VT3989 phase I/II study demonstrated clinical benefit with the YAP/TEAD inhibitor regardless of NF2 mutation status [12]. Nevertheless, somatic testing may retain clinical utility by identifying high variant allele frequency mutations that can suggest an underlying germline origin [13].

Among modifiable barriers, lack of physician awareness—identified by nearly half of respondents—represents the most actionable target. Scientific societies such as MET-I are well positioned to address this through targeted educational initiatives, including webinars, written guidelines, and structured training programs. In addition, nearly a third of survey respondents identify lack of specific guidelines as a major barrier for germline testing implementation in mesothelioma patients. Dedicated scientific societies, such as MET-I, might solve this issue through discussing with guideline panel members the relevance in clinical practice for adequate germline and somatic testing in mesothelioma.

This study has several limitations: respondents were predominantly from Northern Italy and academic centers, the sample size was relatively small, and the survey design provides only a descriptive overview. Nonetheless, these results offer the first real-world snapshot of genetic testing practices in Italian PM care and may inform targeted interventions to improve clinical implementation.

## Data Availability

No datasets were generated or analysed during the current study.
